# COMMUNAL SUPPORT PREDICTS BETTER MENTAL HEALTH: KNOWLEDGE TRANSLATION AMONG OLDER ADULTS DURING COVID-19

**DOI:** 10.1093/geroni/igac059.2304

**Published:** 2022-12-20

**Authors:** Eireann O'Dea, Daniel Rong Yao Gan, Habib Chaudhury, Ziying Zhang, Andrew Wister, Lisa Cohen Quay, Shelley Jorde, Claire Wang

**Affiliations:** Simon Fraser University, North Vancouver, British Columbia, Canada; Simon Fraser University, Vancouver, British Columbia, Canada; Simon Fraser University, Vancouver, British Columbia, Canada; Simon Fraser University, Vancouver, British Columbia, Canada; Simon Fraser University, Vancouver, British Columbia, Canada; Jewish Community Centre of Greater Vancouver, Vancouver, British Columbia, Canada; South Vancouver Seniors Hub, Vancouver, British Columbia, Canada; Johns Hopkins University , Baltimore City, Maryland, United States

## Abstract

The well-being of older adults has been linked to the quality of their neighbourhood environment. Given that COVID-19 affected poorer neighbourhoods disproportionately, we partnered with community organizations to identify meso-level psychosocial factors that may improve loneliness, depressive mood, and cognitive function. Five variables were identified through focus groups with older adults and community organizations. These variables were drawn from validated scales, including communal provisions, neighbourhood friendship, self-expression, social experiences, and time outdoors. This paper presents preliminary findings from surveys administered to 151 community-dwelling older adults across British Columbia and interviews in four neighbourhoods.Purposeful and snowball sampling were used to recruit older adults (age 55+) from community centres and neighbourhood houses. Online surveys measured the five meso-level psychosocial exposure variables. Outcome variables included an index of loneliness, depressive mood, self-rated memory, semantic fluency and delayed recall. Data was geocoded and aggregated by Forward Sortation Area. Regression and cross-level mediation analysis were conducted. Four neighbourhoods were selected from a 2x2 matrix of high and low neighbourhood deprivation (CANUE, 2016). Mental health was associated with better social experiences (B=.26, p=.003). Time outdoors (B=.35, p=.047) was associated with better delayed recall. Mental health was better in poorer neighbourhoods (B=.20, p=.015). This was partially mediated by communal provisions (B=.19, p=.032). Social experiences (B=.23, p=.009) fully mediated these effects on mental health. Participants described being of local community services and took on opportunities to volunteer. Social experiences and neighbourhood resources may help support mental health and well-being among older adults during the pandemic and beyond.

